# Quantitative SPECT/CT for differentiating between enchondroma and grade I chondrosarcoma

**DOI:** 10.1038/s41598-020-67506-4

**Published:** 2020-06-29

**Authors:** Woo Hee Choi, Eun Ji Han, Ki Bong Chang, Min Wook Joo

**Affiliations:** 1 Division of Nuclear Medicine, Department of Radiology, St. Vincent’s Hospital, College of Medicine, The Catholic University of Korea, Seoul, Republic of Korea; 2 Division of Nuclear Medicine, Department of Radiology, Yeouido St. Mary’s Hospital, College of Medicine, The Catholic University of Korea, Seoul, Republic of Korea; 3 Department of Orthopaedic Surgery, St. Vincent’s Hospital, College of Medicine, The Catholic University of Korea, 222, Banpo-daero, Seocho-gu, Seoul, 06591 Republic of Korea

**Keywords:** Cancer, Medical research, Oncology

## Abstract

Although differentiation between central chondroid tumors is important, their parallelism makes it a diagnostic conundrum for clinicians and radiologists. The objective of this study was to evaluate the efficiency of quantitative single photon emission computed tomography (SPECT)/computed tomography (CT) in differentiating grade I chondrosarcomas from enchondromas. We reviewed SPECT/CT images of patients with enchondromas and grade I chondrosarcomas arising in the long bones. Volume, mean standardized uptake value (SUVmean), and maximum standardized uptake value (SUVmax) of tumors were calculated from SPECT/CT images. In addition, clinical characteristics and radiological information were assessed. Of a total of 34 patients, 14 had chondrosarcomas. Chondrosarcoma group had significantly larger volume, and higher SUVmean and SUVmax of tumors than enchondroma group. There was no significant difference in age and tumor size between two groups. Areas under the receiver-operating characteristic curve (AUCs) for tumor volume, SUVmean, and SUVmax were 0.727, 0.757, and 0.875. In pairwise analyses, SUVmax had larger AUC than SUVmean (p = 0.0216). With a cut-off value of 15.6 for SUVmax, its sensitivity and specificity were 86% and 75% for differentiating between enchondroma and grade I chondrosarcoma. Quantitative SPECT/CT is a potential method to differentiate grade I chondroarcomas from enchondromas in patients with central chondroid tumors.

## Introduction

Differentiation between central chondroid tumors is important because surgical treatment is the only curative management for patients with grade I chondrosarcoma. A wait-and-see policy is permissible for enchondromas in consideration of long-term prognosis^1,2^. Nevertheless, their parallelism makes it a diagnostic conundrum for clinicians and radiologists to distinguish them^3^. Clinical symptoms such as spontaneous pain are believed to be helpful in distinction. However, patients with enchondroma uncommonly complained of resting pain and the presentation without symptom does not rule out malignancy^4^. Although poorly defined margin, deep endosteal scalloping, the size over 5 cm, periosteal reaction without a history of trauma, and epiphyseal location in plain radiography favor chondrosarcoma, the discriminating power of this set of parameters is limited^2,5^. While chondrosarcomas with lobulated lesion, osseous destruction and fibrovascular septae on magnetic resonance imaging (MRI), and early enhancement pattern on dynamic contrast-enhanced (DCE) MRI can be used for differential diagnosis of chondroid tumors^6^, their usefulness remains controversial^5,7,8^. Interpretation of radiological findings demonstrated low reliability for grading of cartilaginous tumors, even among expert radiologists^3^. Diffusion-weighted MRI and quantitative assessment by apparent diffusion coefficient values are expected to be attributed to more accurate and consistent diagnosis for that reason. However, the role of diffusion-weighted MRI is also questioned^3,9^. Although F-18 fluorodeoxyglucose positron emission tomography (PET) study has been reported to be a valuable adjunct^10^, controversial reports also exist^10,11^. Histologic diagnosis and grading of cartilaginous tumors are also challenging and subjected to a large inter-observer variability. Host bone entrapment, marked nuclear pleomorphism, high cellularity, and irregular distribution of cells are known as pathologic parameters with the most discriminating strength for differentiation of central grade I chondrosarcoma from enchondroma^4^.

Radionuclide bone imaging visualizes osteoblatic activity related to bone diseases. Bone scintigraphy has been attempted to distinguish between enchondroma and low-grade chondrosarcoma. Cortical destruction and permeation due to the chondroid tumor can be reflected on bone scintigraphy. Chondrosarcomas generally demonstrate marked heterogenous radionuclide uptake with intensity greater than anterior iliac crest whereas enchondromas show increased radionuclide uptake in only a small proportion^7,12^. Nonetheless, traditional imaging using a scintillation camera including single photon emission computed tomography (SPECT) provides non-quantitative information. Recently, advances in technology have allowed a combined modality of SPECT/computed tomography (CT) to measure radiotracer distribution quantitatively as PET/CT does^13^.

To our knowledge, no study has evaluated the utility of quantitative SPECT/CT for differentiation between chondroid tumors in the long bone. Thus, the objective of this study was to investigate the efficiency of quantitative SPECT/CT in differentiating grade I chondrosarcomas from enchondromas.

## Results

A total of 34 patients (12 men and 22 women) were included in this study. Their mean age was 51 ± 14 years (range 19–75 years). Fourteen patients were histologically confirmed as grade I chondrosarcoma after definitive surgery. Clinical characteristics and SPECT/CT findings are described in Table 1. There was no significant difference in age or tumor size between enchondroma and chondrosarcoma groups. About 57% of chondrosarcoma patients and 25% of patients with enchondroma had tumors greater than 5 cm in size, showing no significant difference between two groups (p = 0.133). Patients with grade I chondrosarcoma had a significantly higher incidence of hyperemia (p = 0.035). Tumor volume, mean standardized uptake value (SUVmean), and maximum standardized uptake value (SUVmax) also showed statistically significant differences between two groups. The SUVmax of grade I chondrosarcomas ranged from 10.9 to 78.1 while that of enchondroma from 2.8 to 19.2.

**Table 1:** Clinical characteristics and SPECT/CT findings in patients with enchondroma and grade I chondrosarcoma.

	Enchondroma (n = 20)	Grade I chondrosarcoma (n = 14)	*p* value
Gender			
Male	7 (35%)	5 (36%)	
Female	13 (65%)	9 (64%)	
Age (years)	52 ± 12	49 ± 16	^†^0.612
Skeletal distribution			
Humerus	11	5	
Femur	7	5	
Radius		1	
Tibia		1	
Fibula	2	2	
Tumor size (cm)	4.2 ± 2.4	5.2 ± 1.8	^†^0.119
Hyperemia on blood pool image	5 (25%)	9 (64%)	^‡^0.035*
Tumor volume (cm^3^)	9.7 ± 9.5	14.8 ± 7.6	^†^0.026*
SUVmean	5.8 ± 2.5	9.9 ± 5.9	^†^0.012*
SUVmax	12.4 ± 5.0	24.8 ± 16.5	^†^< 0.001*

^†^Mann–Whitney U test.

^‡^Fisher’s exact test.

*Statistically significant.

Of the 34 patients, 29 performed whole body bone scans before the acquisition of SPECT/CT image. Of these 29 patients who had whole body bone scan, 13 showed higher radionuclide uptake in the chondroid lesions compared to physiologic uptake in the anterior iliac crest (Table 2). Among them, eight were histologically diagnosed as chondrosarcoma. Of 16 cases that demonstrated equal or lower radionuclide uptake, three were confirmed to be chondrosarcoma. There was a significant difference in the incidence of higher radionuclide uptake between enchondroma (3/18) and chondrosarcoma (8/11) groups (p = 0.027).

**Table 2:** Degree of radionuclide uptake in whole body bone scintigraphy.

Radionuclide uptake	Enchondroma (n = 18)	Chondrosarcoma (n = 11)
< Uptake to anterior iliac crest	5	0
= Uptake to anterior iliac crest	8	3
> Uptake to anterior iliac crest	5	8

**Figure 1:**
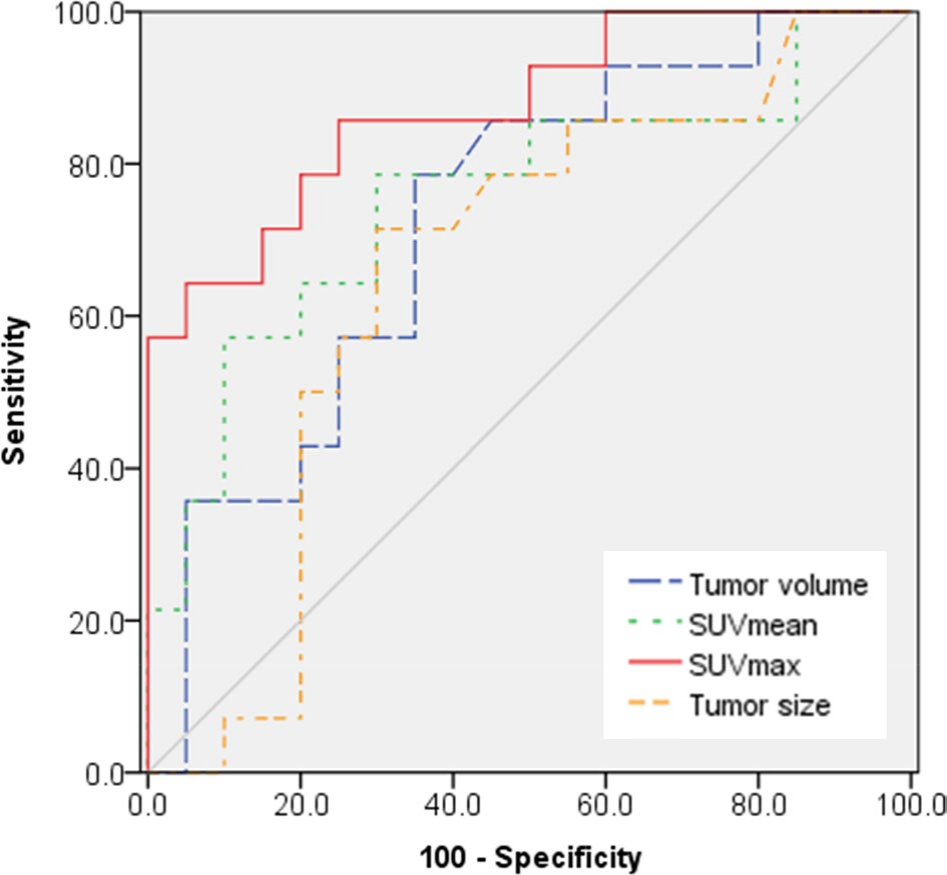
Receiver operating characteristic curves for tumor size, tumor volume, SUVmean, and SUV max for discriminating grade I chondrosarcoma from enchondroma.

Receiver-operating characteristic (ROC) curve analysis showed that the area under the curve (AUC) for SUVmax was the largest [AUC = 0.875; p < 0.001; 95% confidence interval (CI): 0.755–0.995)], followed by AUC for SUVmean (AUC = 0.757; p = 0.012; 95% CI: 0.582–0.932) and tumor volume (AUC = 0.727, p = 0.026; 95% CI: 0.555–0.899) (Fig. 1). ROC curve for tumor size showed an AUC of 0.659. However, it showed no significant difference from the line of reference (p = 0.119; 95% CI: 0.468–0.850). In pairwise analyses, SUVmax had larger AUC than SUVmean (p = 0.0216) (Table 3). Using ROC analysis, the best cut-off point was found to be 7.3 for SUVmean and 15.6 for SUVmax with which the maximum sensitivity and specificity for discriminating enchondroma and grade I chondrosarcoma were obtained. Sensitivity, specificity, positive predictive value (PPV), negative predictive value (NPV), and accuracy for imaging parameters are summarized in Table 4. With a cut-off value of 15.6 for SUVmax, its sensitivity and specificity were 86% and 75%, respectively in differentiating between enchondroma and grade I chondrosarcoma.

**Table 3:** Pairwise comparison of ROC curves.

	Difference between areas	Standard error	95% confidence interval	Z statistics	p value
SUVmax − SUVmean	0.113	0.0490	0.0165–0.209	2.297	0.0216*
SUVmax − Volume	0.146	0.119	− 0.0873 to 0.380	1.228	0.2194
SUVmean − Volume	0.0339	0.143	− 0.246 to 0.313	0.238	0.8120

*Statistically significant.

**Table 4:** Diagnostic performance in differentiating enchondroma and grade I chondrosarcoma.

	Sensitivity	Specificity	Positive predictive value	Negative predictive value	Accuracy
Hyperemia	64.3	75.0	64.3	75.0	70.6
Higher radionuclide uptake	72.7	72.2	61.5	81.3	72.4
Tumor size (> 5 cm)	50.0	75.0	58.3	68.1	64.7
Tumor volume (> 7.9 cm^3^)	78.6	65.0	61.1	81.3	70.6
SUVmean (> 7.3)	78.6	70.0	64.7	82.4	73.5
SUVmax (> 15.6)	85.7	75.0	70.6	88.2	79.4

## Discussion

Tumor length has conventionally been regarded as one of radiological parameters for differentiation between enchondroma and grade I chondrosarcoma^2,5^. However, some previous reports suggested that large chondroid lesions without a permeative growth pattern should be termed separately as cartilaginous lesion of unknown malignant potential or grade 0 chondrosarcoma regardless of lesion size^14,15^. While a recent study has also reported that tumor size can suggest the presence of chondrosarcoma as opposed to enchondroma, it emphasizes that its sensitivity is low in grade I chondrosarcoma^8^. Results of the present study revealed that tumor length over 5 cm was not a statistically significant parameter for distinguishing between enchondroma and grade I chondrosarcoma.

On the contrary, grade I chondrosarcoma group had significantly larger volume than enchondroma group in this study. Because a solid tumor is a three-dimensional structure which grows at non-uniform rate in each direction, the main length may not reflect the tumor characteristics in detail. Therefore, we believe that tumor volume would be a better parameter to assess tumor aggressiveness.

Hyperemia on blood pool image was more common in chondrosarcoma group of this study. Blood pool image indicates increased retention of the radiotracer in the soft tissues due to increased vascular permeability or neovascularity^16,17^. A previous study has reported higher pericartilage neovascularization in chondrosarcoma than that in enchondroma^18^. However, the presence of hyperemia on blood pool image showed insufficient diagnostic performance in differentiating grade I chondrosarcoma from enchondroma, reflecting the limited use of vascularity in distinguishing between these lesions.

Chondrosarcomas typically demonstrate high radionuclide uptake on bone scintigraphy. Murphey et al.^19^ have reported that 82% of chondrosarcomas show greater radionuclide uptake than the anterior iliac crest on whole body bone scintigraphy whereas only 21% of enchondromas demonstrate greater uptake. Similarly, 73% of chondrosarcoma and 28% of enchondroma showed higher uptake than the anterior iliac crest in the present study. This might reflect higher degree of reaction in surrounding bone that causes greater radionuclide uptake such as cortical extension or permeation in chondrosarcoma. However, visual grading of radionuclide uptake on bone scintigraphy is subjective and prone to inter-reader variability, although it is an easy and common method. Furthermore, the distance between the detector of camera and tumor may affect visual assessment. Inappropriate positioning or rotation of the pelvis may also make it difficult to draw accurate judgements.

**Figure 2:**
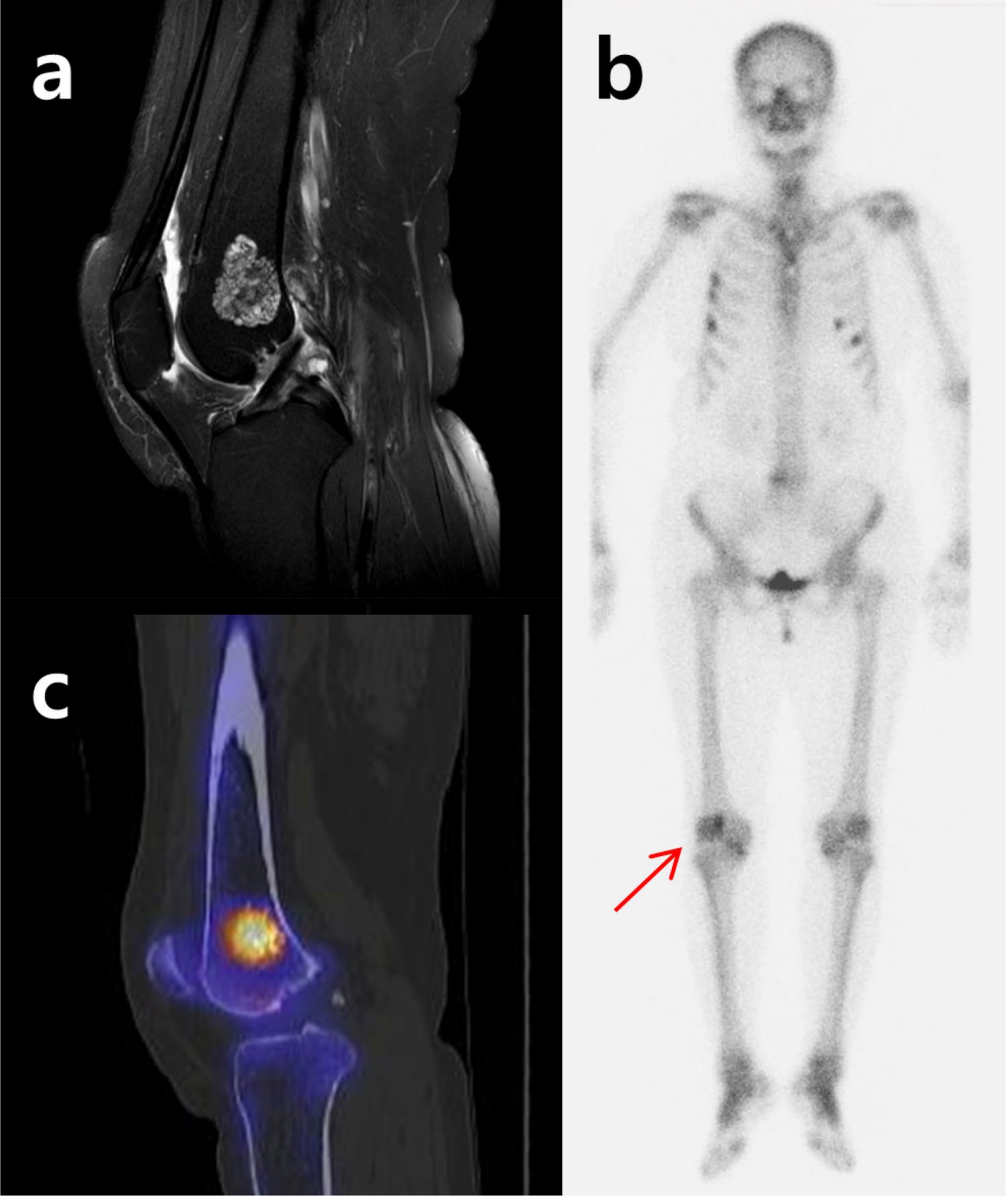
A 76-year-old female patient who was diagnosed with enchondroma 9 years ago. (**a**) Follow-up MRI shows no interval change. On fat-suppressed contrast-enhanced T1-weighted images, about 3.2 cm sized mass with nodular and septal enhancement in distal metaphysis of femur is shown. (**b**) Whole body bone scintigraphy demonstrates focal radionuclide uptake on right distal femur (arrow) which has higher uptake than anterior iliac crest. (**c**) On the SPECT/CT images, the SUVmax of the lesion was calculated to be 14.7.

**Figure 3:**
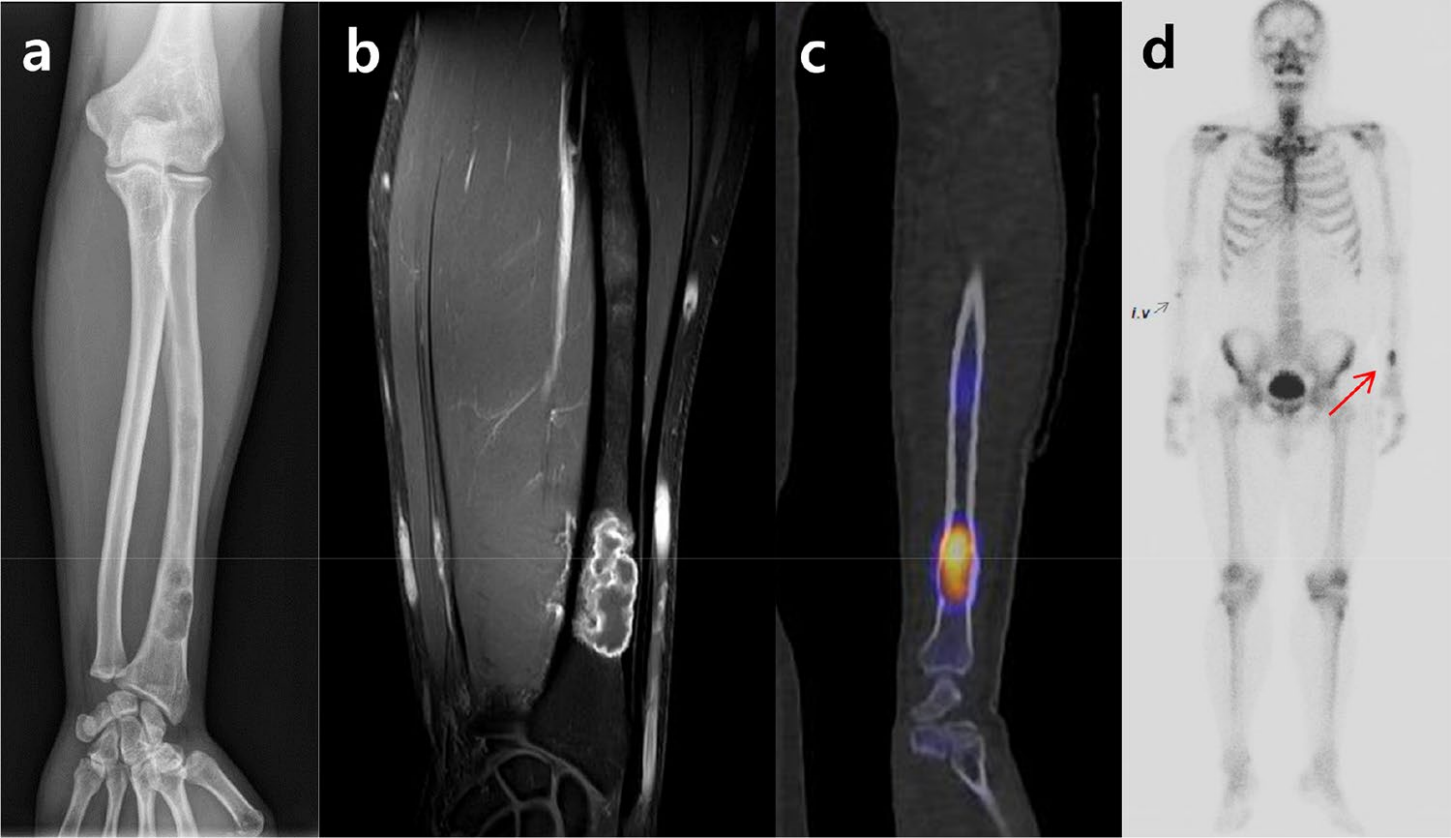
A 35-year-old male patient who was diagnosed grade I chondrosarcoma. (**a**) A geographic osteolytic lesion with cortical erosion was incidentally detected at metaphysis of left distal radius. (**b**) On fat-suppressed contrast-enhanced T1-weighted images, a 3.3 cm sized lobulated mass with rim and septal enhancement and endosteal scalloping involving more than two third of cortical thickness is shown. (**c**) On SPECT/CT images, the SUVmax of this lesion was calculated to be 22.9. (**d**) Whole body bone scan image shows focal radionuclide uptake equal to the anterior iliac crest in left distal radius (arrow).

SUVmax is a feasible semi-quantitative parameter that frequently used for assessment of radiotracer accumulation, especially in PET/CT. It is now available for SPECT/CT. SPECT/CT imaging reports quantitative accuracy within 5% of the true radionuclide concentration. This is equivalent to the accuracy of current PET/CT systems^13,20^. By using SUVmax, diagnostic accuracy of bone imaging with Tc-99 m labeled diphosphonates is improved (Figs. 2, 3). In the present study, the SUVmax of grade I chondrosarcomas ranged from 10.9 to 78.1 while that of enchondroma range ranged from 2.8 to 19.2. Even if the best cut-off using Youden index which is the cut-point (15.6 in the present study) that equal weight is given to sensitivity and specificity, careful observation should be considered in the patient with SUVmax within a grey zone because it is even more essential not to miss chondrosarcoma, although it is also important to avoid unnecessary biopsy.

Tumor size is an important factor which affects standardized uptake values (SUVs) in SPECT/CT^21,22^. If the diameter of a lesion is less than two or three times the full width at half maximum of the reconstructed image resolution, the SUVs are subject to partial-volume effect and can be measured lower than the real values. In this study, the tumor size seemed to have little effect on the measurement accuracy because the diameters were larger than three times the system spatial resolution in most tumors^21,22^. Only one lesion was a 1.1 cm sized enchondroma of which the SUVmax was calculated as 2.84.

Besides tumor size, several factors can affect the SUVs in bone SPECT/CT. Age seems to have an influence on SUVs^23,24^. In addition, bone mineral density may be positively correlated with SUVs^23,24^. On the contrary, there is still controversy over the impact of other factors. SUVs in the normal vertebrae did not show a significant correlation with subject’s weight but with the height in a study^25^; however, another research conveyed the opposite^24^. Regarding differences in SUVs by gender, previous studies also showed different results^25,26^.

Recently, a theoretical background for DCE MRI to distinguish benign from malignant cartilage tumors compared to standard MRI has been suggested as it obtains information of tumor vascularization and perfusion^6^. However, the information is not a histologic differential point between enchondroma and grade I chondrosarcoma^4^. We believe that quantitative measure on osteoblastic activity by SPECT/CT should superiorly reflect biological feature of grade I chondrosarcoma based on histologic feature of permeative growth pattern^4,8^. Moreover, the DCE MRI images are acquired in only one imaging plane so that information can be measured in the selected area^6^.

Over the past few years, intralesional procedures have been advocated more often in the literature than wide excision in the management of grade I chondrosarcoma^27^. Extended curettage followed by adjuvant treatments is currently a definitive treatment in many institutions as well as ours. Accordingly, it is difficult to obtain specimens of en bloc resection. In addition, whole lesions were not also examined pathologically in enchondroma cases because definitive surgery is not mandatory for them. Therefore, we could not analyze the relationship between SPECT/CT quantitative parameters and pathologic features in each region of tumors although we believe that focal presence of host bone entrapment could increase osteoblastic activity in the affected region and consequently raise the local SUV and the SUVmax of the tumors in SPECT/CT. Such current clinical situation should be considered in additional well-designed research.

This study could exclude diagnostic review bias which usually occurs as interpretation of histologic findings is achieved with knowledge of imaging results or clinical data in previous literature^28^ since no one in diagnostic multidisciplinary team had information from quantitative SPECT/CT. However, incorporation bias could not be avoided as histologic diagnosis was used to validate the criteria from SPECT/CT. Moreover, there was a possibility of inclusion bias due to small number of cases and non-representative patient selection. To prevent such systematic biases, the most definite method is to choose outcome analysis as a gold standard for distinction of chondroid tumors rather than histologic diagnosis. Although it is difficult to use oncologic outcome to confirm the diagnosis of grade I chondrosarcomas because of a low recurrence and metastasis rates, we believe that additional evaluation on efficiency of quantitative SPECT/CT according to the outcome is needed.

Quantitative SPECT/CT is a potential method to differentiate grade I chondrosarcomas from enchondromas in patients with central chondroid tumors. Further studies with more cases and longer follow-up period are needed to validate this result.

## Methods

### Patients

Patients with enchondroma and grade I chondrosarcoma arising in the long bones who had bone SPECT/CT between December 2015 and October 2018 were included in this study. The Catholic University of Korea St. Vincent’s Hospital Institutional Review Board approved this research (VC18RESI0203) and waived the need for written informed consent because this retrospective review was a minimal risk study and no personally identifiable information was collected. The work was performed in accordance with the relevant guidelines and regulations of the ethical committee. The need for written informed consent was waived by the approved study protocol because this was a retrospective review and minimal risk study, and we did not collect any personally identifiable information. Inclusion criteria for enchondroma were tumors originating within bone and histological diagnosis based on biopsy specimen. Inclusion criteria for grade I chondrosarcoma were tumors developing within bone and pathological diagnosis based on the specimen from a biopsy or definitive surgery. Patients who underwent bone SPECT/CT scan only after surgical removal or biopsy were excluded. Clinical and radiological information such as age, gender, tumor location, and imaging findings were obtained from medical records.

### Image acquisition

All SPECT/CT studies were obtained using a SPECT/CT scanner, NMCT/670 (GE Healthcare, Waukesha, WI, USA). Planar blood pool imaging was performed at 1 min after intravenous injection of 800–1,100 MBq of Tc-99 m hydroxymethylene diphosphonate. Four hours after injection of radiotracer, whole body image and planar spot image of the site of tumor were obtained followed by SPECT/CT. CT images were acquired by using the following parameters: 120 kVp, 110–130 mA with “autoMa” function, and 1.25-mm-thick sections. SPECT images were acquired by using the following parameters: peak energy at 140 keV with 10% window and step-and-shot mode acquisition (25 s per step and 30 steps per detector) with 6° angular increment. SPECT images reconstructions were performed using an iterative ordered subset expectation maximization algorithm (four iterations; 10 subsets) with CT-based attenuation correction, scatter correction, and resolution recovery on a Xeleris imaging workstation (version 4.0, GE Healthcare, Waukesha, WI, USA). The matrix size of reconstructed images was 128 × 128 with section thickness of 4.42 mm. The minimal source-to-collimator distance is 4 mm for parallel-hole collimation of Tc-99 m.

### Image analysis

All images were evaluated by an experienced nuclear medicine physician blinded to histologic result. The presence of hyperemia was visually evaluated based on blood pool image. On the whole-body image, the degree of radionuclide uptake in the lesion was compared with that in anterior iliac crest. If radionuclide uptake in the lesion was equal to or higher than that of the anterior iliac crest, the case was regarded to have high osteoblastic activity.

All SPECT/CT images were closely evaluated on a dedicated workstation (Xeleris 4.0, GE Healthcare, Waukesha, WI, USA) that displayed CT, SPECT, and fused SPECT/CT images. For quantitative analysis, the regions of interest (ROIs) were manually drawn over the chondroid tumors on each slice of SPECT/CT by an experienced nuclear medicine physician who reviewed magnetic resonance images. Then, the final volumes of interest (VOIs) were generated by integrating multiple ROIs. Quantitative parameters were obtained from VOIs using the Q.Metrix toolkit installed on the dedicated workstation. SUVmean and SUVmax in a given VOI were calculated as follows:

$$\begin{aligned} & {\text{SUVmean}}\, = \,\left( {{\text{total}}\,{\text{radioactivity/VOI}}\,{\text{volume}}} \right)/\left( {{\text{injected}}\,{\text{radioactivity/body}}\,{\text{weight}}} \right)\,\,\,\left( {\text{g/ml}} \right) \\ & {\text{SUVmax}}\, = \,\left( {{\text{maximum}}\,{\text{radioactivity/voxel}}\,{\text{volume}}} \right)/\left( {{\text{injected}}\,{\text{radioactivity/body}}\,{\text{weight}}} \right)\,\,\,\left( {\text{g/ml}} \right) \\ \end{aligned}$$

In addition, the longest diameter of each tumor was measured on MRI.

### Statistics

Categorical variables are expressed as numbers and percentages while continuous variables are presented as mean and standard deviation. Enchondroma and chondrosarcoma groups were compared using Mann–Whitney U test for continuous variables and Fisher’s exact test for categorical variables. A ROC curve analysis was performed to evaluate the diagnostic performance. The AUC was calculated. The cut-off value for differentiating the two groups was determined as the values corresponding to the maximum of the Youden index. Sensitivity, specificity, accuracy, PPV, NPV, and accuracy were calculated. The overall comparison of diagnostic performance was derived from differences between AUCs. All statistical analyses were performed using the Statistical Package for the Social Sciences software (version 21, IBM Corp., Armonk, NY, USA) and MedCalc software (version 10.1.3.1, MedCalc Inc., Mariakerke, Belgium). P values < 0.05 were considered statistically significant.

## Data Availability

All data generated or analyzed during this study are included in this published article.
